# Circadian nutritional behaviours and risk of type 2 diabetes in NutriNet-Santé

**DOI:** 10.1093/eurpub/ckac129.492

**Published:** 2022-10-25

**Authors:** B Srour, A Palomar-Cros, VA Andreeva, LK Fezeu, C Julia, A Bellicha, E Kesse-Guyot, D Romaguera, M Kogevinas, M Touvier

**Affiliations:** 1 EREN, Inserm, Inrae, Cnam, USPN, INSERM, Bobigny, France; 2 Barcelona Institute for Global Health, ISGlobal, Barcelona, Spain; 3 Avicenne Hospital, Public Health Department, Bobigny, France; 4 IdISBa, Palma de Mallorca, Spain; 5 CIBEROBN, Madrid, Spain; 6 IMIM, Barcelona, Spain; 7 CIBERESP, Madrid, Spain

## Abstract

Skipping breakfast and late-night-eating have been associated with risk factors for type 2 diabetes (T2D). However, less is known about the link between daily timing and frequency of food intake and risk of developing T2D. The objective of the present study is to investigate the associations between circadian nutritional behaviours, defined by meal timings and frequency, and risk of T2D. 103,312 adults (79% females, mean age at baseline=42.7) from the French NutriNet-Santé cohort were included. Participants’ circadian nutritional behaviours were assessed using repeated 24 h dietary records. Associations of time of first and last meal of the day, meal frequency and of nighttime fasting duration with risk of T2D were assessed by multivariable Cox proportional hazard models adjusted for known risk factors. During a median follow-up of 7.3 years, 963 new cases of T2D were ascertained. Compared with subjects reporting on average a first meal before 8AM, those having a first meal after 9AM had a higher risk of developing T2D, HR = 1.59 (1.30 to 1.94). A late time of last meal (after 9PM) was associated with a higher risk of T2D, HR = 1.28 (1.06 to 1.54), but this association was no longer significant after adjusting for time of first meal. Each additional eating episode was associated with a reduction of the risk of T2D, HR = 0.95 (0.90 to 0.99), p-value=0.01. Overall, nighttime fasting duration was not associated with risk of T2D, except in participants having breakfast before 8AM after a nighttime fasting duration of more than 13 hours (HR = 0.47, 0.27 to 0.82). In this large prospective study, circadian nutritional behaviours were associated with risk of T2D. Daytime nutritional behaviours and specifically an early first meal was associated with a lower risk of type 2 diabetes. If confirmed in other largescale studies, an early breakfast should be considered in preventive strategies for type 2 diabetes.

**Key messages:**

• If confirmed in other largescale studies, an early breakfast could be considered in preventive strategies for type 2 diabetes.

• Beyond nutritional quality of meals, meal timing could also be a risk factor for type-2 diabetes.

